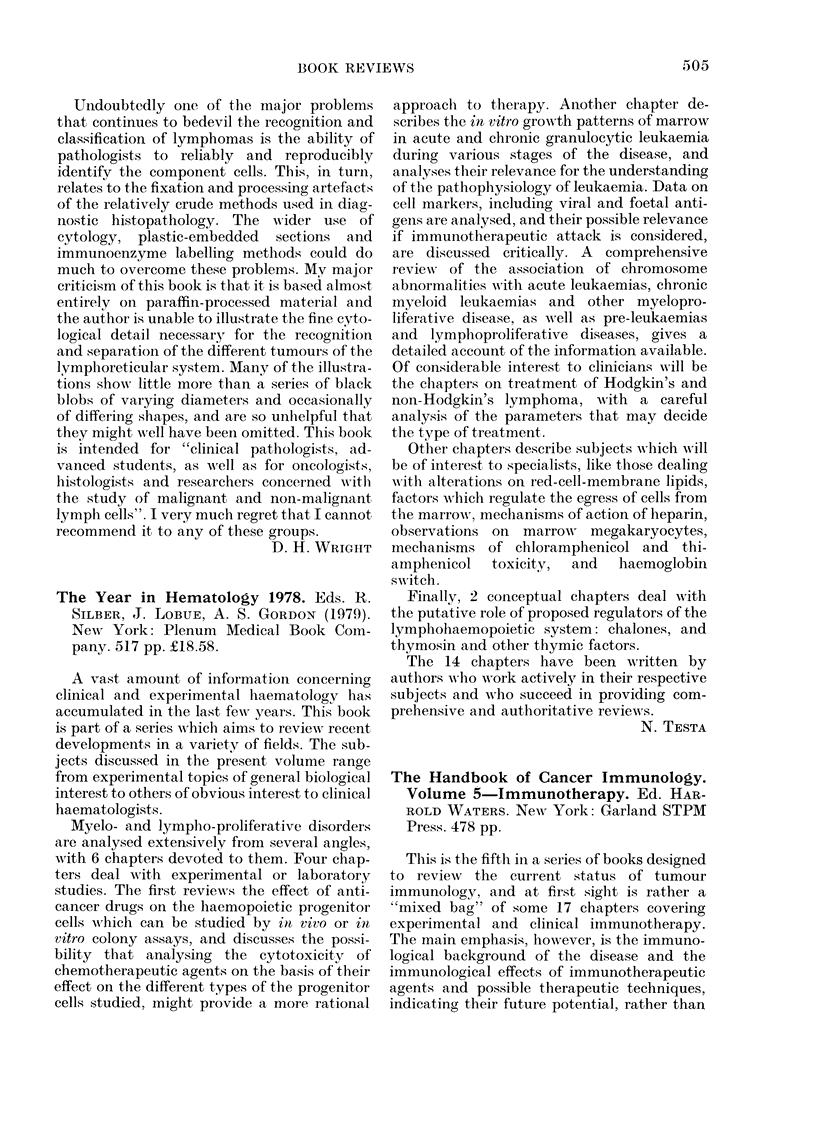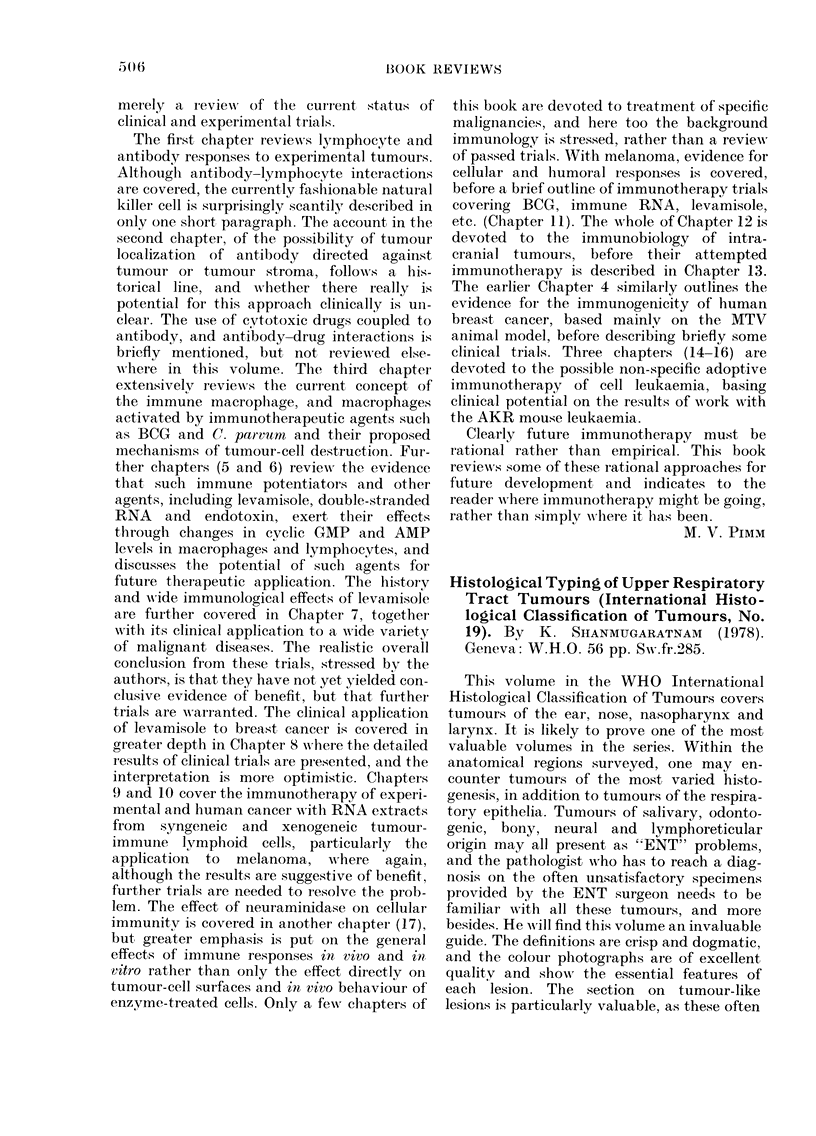# The Handbook of Cancer Immunology. Volume 5—Immunotherapy

**Published:** 1979-09

**Authors:** M. V. Pimm


**The Handbook of Cancer Immunology. Volume 5—Immunotherapy.** Ed. Harrold Waters. New York: Garland STPM Press. 478 pp.

This is the fifth in a series of books designed to review the current status of tumour immunology, and at first sight is rather a "mixed bag" of some 17 chapters covering experimental and clinical immunotherapy. The main emphasis, however, is the immunological background of the disease and the immunological effects of immunotherapeutic agents and possible therapeutic techniques, indicating their future potential, rather than merely a review of the current status of clinical and experimental trials.

The first chapter reviews lymphocyte and antibody responses to experimental tumours. Although antibody–lymphocyte interactions are covered, the currently fashionable natural killer cell is surprisingly scantily described in only one short paragraph. The account in the second chapter, of the possibility of tumour localization of antibody directed against tumour or tumour stroma, follows a historical line, and whether there really is potential for this approach clinically is unclear. The use of cytotoxic drugs coupled to antibody, and antibody–drug interactions is briefly mentioned, but not reviewed elsewhere in this volume. The third chapter extensively reviews the current concept of the immune macrophage, and macrophages activated by immunotherapeutic agents such as BCG and *C. parvum* and their proposed mechanisms of tumour-cell destruction. Further chapters (5 and 6) review the evidence that such immune potentiators and other agents, including levamisole, double-stranded RNA and endotoxin, exert their effects through changes in cyclic GMP and AMP levels in macrophages and lymphocytes, and discusses the potential of such agents for future therapeutic application. The history and wide immunological effects of levamisole are further covered in Chapter 7, together with its clinical application to a wide variety of malignant diseases. The realistic overall conclusion from these trials, stressed by the authors, is that they have not yet yielded conclusive evidence of benefit, but that further trials are warranted. The clinical application of levamisole to breast cancer is covered in greater depth in Chapter 8 where the detailed results of clinical trials are presented, and the interpretation is more optimistic. Chapters 9 and 10 cover the immunotherapy of experimental and human cancer with RNA extracts from syngeneic and xenogeneic tumour-immune lymphoid cells, particularly the application to melanoma, where again, although the results are suggestive of benefit, further trials are needed to resolve the problem. The effect of neuraminidase on cellular immunity is covered in another chapter (17), but greater emphasis is put on the general effects of immune responses *in vivo* and *in vitro* rather than only the effect directly on tumour-cell surfaces and *in vivo* behaviour of enzyme-treated cells. Only a few chapters of this book are devoted to treatment of specific malignancies, and here too the background immunology is stressed, rather than a review of passed trials. With melanoma, evidence for cellular and humoral responses is covered, before a brief outline of immunotherapy trials covering BCG, immune RNA, levamisole, etc. (Chapter 11). The whole of Chapter 12 is devoted to the immunobiology of intracranial tumours, before their attempted immunotherapy is described in Chapter 13. The earlier Chapter 4 similarly outlines the evidence for the immunogenicity of human breast cancer, based mainly on the MTV animal model, before describing briefly some clinical trials. Three chapters (14–16) are devoted to the possible non-specific adoptive immunotherapy of cell leukaemia, basing clinical potential on the results of work with the AKR mouse leukaemia.

Clearly future immunotherapy must be rational rather than empirical. This book reviews some of these rational approaches for future development and indicates to the reader where immunotherapy might be going, rather than simply where it has been.

M. V. PIMM